# Large-scale pedigree analysis highlights rapidly mutating Y-chromosomal short tandem repeats for differentiating patrilineal relatives and predicting their degrees of consanguinity

**DOI:** 10.1007/s00439-022-02493-2

**Published:** 2022-10-03

**Authors:** Arwin Ralf, Diego Montiel González, Dion Zandstra, Bram van Wersch, Nefeli Kousouri, Peter de Knijff, Atif Adnan, Sofie Claerhout, Mohsen Ghanbari, Maarten H. D. Larmuseau, Manfred Kayser

**Affiliations:** 1 Department of Genetic Identification, Erasmus MC, University Medical Center Rotterdam, Rotterdam, The Netherlands; 2 Forensic Laboratory for DNA Research, Department of Human Genetics, Leiden University Medical Centre, Leiden, The Netherlands; 3 Department of Forensic Sciences, College of Criminal Justice, Naif Arab University of Security Sciences, Riyadh, Saudi Arabia; 4 Forensic Biomedical Sciences, Department of Imaging and Pathology, KU Leuven, Leuven, Belgium; 5 Interdisciplinary Research Facility Life Sciences, KULAK Campus Kortrijk, Kortrijk, Belgium; 6 Department of Epidemiology, Erasmus MC, University Medical Center Rotterdam, Rotterdam, The Netherlands; 7 Laboratory of Human Genetic Genealogy, Department of Human Genetics, KU Leuven, Leuven, Belgium; 8 ARCHES-Antwerp Cultural Heritage Sciences, Faculty of Design Sciences, University of Antwerp, Antwerp, Belgium; 9 Histories vzw, Ghent, Belgium

## Abstract

Rapidly mutating Y-chromosomal short tandem repeats (RM Y-STRs) were suggested for differentiating patrilineally related men as relevant in forensic genetics, anthropological genetics, and genetic genealogy. Empirical data are available for closely related males, while differentiation rates for more distant relatives are scarce. Available RM Y-STR mutation rate estimates are typically based on father–son pair data, while pedigree-based studies for efficient analysis requiring less samples are rare. Here, we present a large-scale pedigree analysis in 9379 pairs of men separated by 1–34 meioses on 30 Y-STRs with increased mutation rates including all known RM Y-STRs (RMplex). For comparison, part of the samples were genotyped at 25 standard Y-STRs mostly with moderate mutation rates (Yfiler Plus). For 43 of the 49 Y-STRs analyzed, pedigree-based mutation rates were similar to previous father–son based estimates, while for six markers significant differences were observed. Male relative differentiation rates from the 30 RMplex Y-STRs were 43%, 84%, 96%, 99%, and 100% for relatives separated by one, four, six, nine, and twelve meioses, respectively, which largely exceeded rates obtained by 25 standard Y-STRs. Machine learning based models for predicting the degree of patrilineal consanguinity yielded accurate and reasonably precise predictions when using RM Y-STRs. Fully matching haplotypes resulted in a 95% confidence interval of 1–6 meioses with RMplex compared to 1–25 with Yfiler Plus. Our comprehensive pedigree study demonstrates the value of RM Y-STRs for differentiating male relatives of various types, in many cases achieving individual identification, thereby overcoming the largest limitation of forensic Y-chromosome analysis.

**Supplementary Information:**

The online version contains supplementary material available at 10.1007/s00439-022-02493-2.

## Introduction

The first short tandem repeat marker from the non-recombining part of the human Y-chromosome (Y-STR) was identified 30 years ago and immediately used in a forensic application (Roewer et al. 1992; Roewer and Epplen 1992). A few years later, more Y-STRs followed (Kayser et al. 1997) and the applications expanded to additional areas such as anthropology, genealogy, and population history. The ability to obtain a male-specific STR profile from DNA mixtures that contain an excess of female DNA, such as commonly confronted with in cases of sexual assault involving a male perpetrator and a female victims, was recognized soon after Y-STRs were introduced to forensic genetics (Prinz et al. 1997) and led to the widespread use of Y-STRs in forensic casework within limited time (Kayser 2017), making forensic genetics one of the major areas of Y-STR application until today. Mutation rate studies of Y-STRs using father–son pairs (Kayser and Sajantila 2001) demonstrated that Y-STRs have similarly moderate mutation rates—generally in the order of one or a few mutations every 1000 generation per locus, as had been established earlier for their autosomal counterparts (Brinkmann et al. 1998). Such relatively low mutation rates explain why in the absence of recombination, male relatives typically share the same Y-STR haplotype, which in forensic applications is disadvantageous. A match between a standard Y-STR haplotype, consisting of markers with moderate mutation rates, of a male suspect and that of a crime scene sample means that the crime scene sample could have originated from the male suspect, or, with the same statistical evidence, from any of his close or distant paternal relatives sharing the same Y-STR haplotype (Ballantyne and Kayser 2012). Hence, it is up to tactical police investigation to establish, by excluding all of his paternal male relatives, that the matching suspect was indeed the likely sample donor.

On the other hand, Y-STR haplotype sharing is advantageous in other areas of Y-STR applications such as when conducting genetic genealogical research (Calafell and Larmuseau 2017). For example, a highly divergent haplotype may indicate a discrepancy between the biological pedigree structures and legal family records (Larmuseau et al. 2019), while shared haplotypes can confirm the biological validity of such records. However, the general lack of Y-haplotype variation within patrilineal relatives also poses limitations to genetic genealogy; for example, low precision when estimating the level of relatedness based on two similar haplotypes (King and Jobling 2009). Y-STRs are also be used in anthropological genetics, e.g., to gain understanding in population substructure (Xu et al. 2015), to trace migration patterns (Cai et al. 2011), or to detect founder effects (Myres et al. 2011). In some of these anthropological applications, Y-STR haplotype sharing between unrelated males is advantageous as it indicates recent shared ancestry, which helps answering questions in population history.

The relatively low number of Y-STRs and the high haplotype resemblance within various Y-SNP based haplogroups due to radiation leads to a relatively high number of shared Y-STR haplotype between unrelated males (identity by state, IBS) especially with the earlier Y-STR kits (de Knijff 2022; Larmuseau et al. 2014). Recently, by continuously increasing the number of Y-STRs in the next generation of commercial Y-STR kits, the IBS problem became smaller and paternal lineage identification gained specificity. However, because most Y-STRs included in commercial kits have moderate mutation rates of a few mutations in 1000 generations per locus, Y-STR haplotype sharing between related men remains a major problem of these kits.

A turning point was marked by the findings of a large-scale Y-STR mutation rate study (Ballantyne et al. 2010) that besides providing mutation rate estimates for 186 Y-STRs in close to 2000 father–son pairs, identified 13 Y-STRs with remarkably high mutation rates, exceeding 10^–2^ mutations per generation (mpg), which were termed rapidly mutating Y-STRs (RM Y-STRs) (Ballantyne et al. 2012). These and subsequent studies demonstrated that RM Y-STRs strongly increase the differentiation of paternally related males compared to standard Y-STRs because of their increased mutation rates (Adnan et al. 2016). Moreover, RM Y-STRs were also shown to improve the differentiation of unrelated males compared to AmpFLSTR™ Yfiler™ PCR Amplification Kit, the state-of-the-art commercial Y-STR testing kit at that time (Ballantyne et al. 2014). As a result of these scientific developments, industry picked-up on these findings and included some (but not all at the time known) RM Y-STRs in their next generation commercial Y-STR kits such as the Yfiler™ Plus PCR Amplification Kit (in the following referred to as Yfiler Plus) (Gopinath et al. 2016) and the PowerPlex Y23 System (Thompson et al. 2013).

Recently, more RM Y-STRs were discovered that further improved the male relative differentiation rates and further increased the advantage over standard Y-STRs in differentiating paternally related men (Ralf et al. 2020). Subsequently, a new genotyping method named RMplex was developed to analyze a total of 30 Y-STRs with increased mutation rates including all 26 currently known RM Y-STRs (Ralf et al. 2021). Most recently, a father–son pair study involving ~ 500 pairs (Neuhuber et al. 2022) demonstrated that RMplex is highly effective and allows to differentiate fathers from their sons in over 40% of the cases and, albeit based on a more limited dataset, 62% of brother pairs. In comparison, the current state-of-the-art commercial Y-STR kit Yfiler™ Plus achieved differentiation in only 13% of the father–son pairs and 33% of the brother pairs in the same samples (Neuhuber et al. 2022). However, data on how these 30 RMplex Y-STRs differentiate more distantly related males is lacking completely thus far as empirical studies in more distantly related males such as from pedigree studies are not available as of yet.

Up to now, knowledge on mutation rates and male relative differentiation rates of RM Y-STRs was mostly established in father–son pair studies (Ballantyne et al. 2010, 2014; Burgarella and Navascués 2011; Ralf et al. 2020; Yuan et al. 2019; Zhang et al. 2017), which in principle only allow for the estimation of how closely related males can be differentiated. Pedigree studies, on the other hand, have the advantage that a broad range of male relationships can be studied and a large number of meiotic divisions can be covered by analyzing only a small number of male samples. This makes such pedigree studies more efficient in reaching the large numbers of meioses needed to establish reliable mutation rate estimates (Boattini et al. 2016, 2019; Claerhout et al. 2018). Mutation rates estimated from pedigree studies come, however, with more uncertainties than those from father–son pair studies, which needs to be considered. On the other hand, for investigating male relative differentiation, pedigree studies have a clear advantage over father–son pair studies because they include both closely and distantly related males. The more men that can be genotyped and the deeper the pedigrees are rooted; the more types of distantly related males are available.

Here, for the first time, we performed a large-scale pedigree study on RM Y-STRs by analyzing 1793 males belonging to a total of 403 pedigrees from three cohort studies of diverse bio-geographic ancestries, allowing for a total of 9379 pairwise comparisons of closely and distantly related men separated by 1–34 generations. We genotyped 30 Y-STRs with increased mutation rates, including all currently known RM Y-STRs, using the RMplex genotyping method. Most of the relative pairs were additionally genotyped the current state-of-the-art commercial Yfiler Plus Kit consisting of mostly moderately mutating Y-STRs, to allow the direct comparison of between Y-STRs with increased mutation rates included in RMplex and those with moderate mutation rates (Yfiler Plus). We estimated male relative differentiation rates for all degrees of relationships based on RMplex and for comparison also for Yfiler Plus. Moreover, we estimated the mutation rates of all 49 Y-STRs we analyzed with both assays and compared them with previous mutation rate estimates established from father–son pairs. Finally, we developed machine-learning based models (i.e., multilayer perceptron classifiers) using simulated data to predict the degree of patrilineal consanguinity based on differences in the Y-STR haplotypes of two related males, and validated them using the empirical data from RMplex and Yfiler Plus obtained in this study.

## Results

### Mutation rates

In this study, three cohorts were analyzed, these cohorts consist of pedigrees characterized by different depths of rooting, different sample sizes, different demographic characteristics, and different biogeographic ancestries. The pedigree-based mutation rates were estimated per each cohort separately and for all three cohorts combined (Table S1). For the Yfiler Plus specific loci, only Cohort 1 was included, as the individuals from the other two cohorts had not been genotyped for that assay. The pedigree-based mutation rates were compared to father–son based consensus mutation rate reference values, which were recently published based on multiple father–son based studies (Neuhuber et al. 2022) (Table S1).

**Fig. 1:**
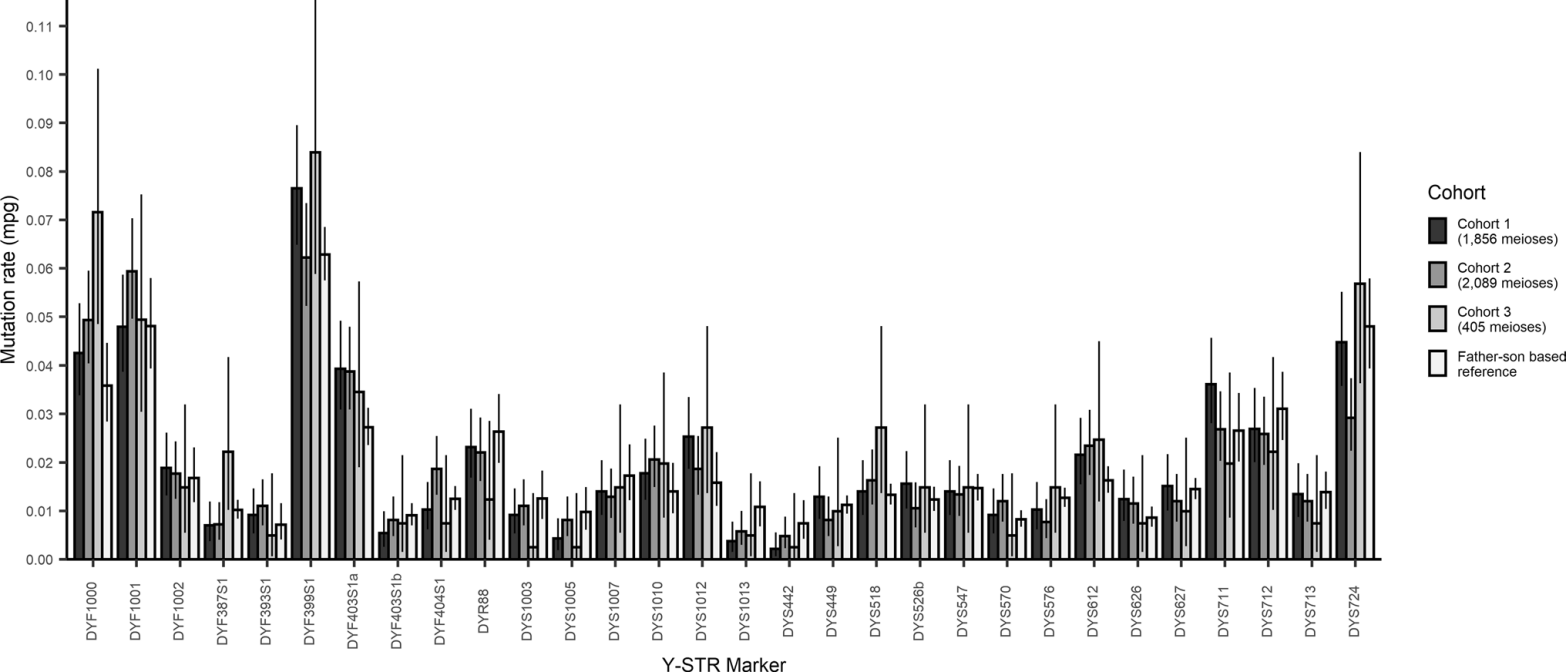
Pedigree-based mutation rate estimates for 30 RMplex Y-STRs from three cohorts as well as the father–son based reference consensus estimates (based on 2025–12,387 meioses per Y-STR) from a recent study (Neuhuber et al. 2022). The error bars represent the 95% Clopper–Pearson intervals

For the vast majority of 43 of the 49 Y-STRs analyzed in total, the obtained pedigree-based mutation rates were coherent with the father–son based mutation rates previously established for these markers. Six Y-STRs showed significant differences between the two ways of estimating mutation rates: DYF1000, DYF403S1a, DYS612, DYS1013, DYS442 and DYS448 (Table S1). For three of those i.e., DYS1000, DYF403S1 and DYS612, the pedigree-based mutation rate estimates were significantly higher than the father–son based rates (*p* value 0.001–0.018). Differences in mutation rate estimates were also found between the three different cohorts (Fig. 1, Table S1), although the overall trends appeared rather consistent across the total pedigree dataset. Notable cohort specific outliers were DYF1000, DYF387S1 and DYS518, which showed remarkably high mutation rates in Cohort 3 consisting of Pakistani males. On the other hand, Cohort 2, which consisted of European males and is characterized by its deep rooting structure, showed a markedly lower mutation rate estimate for DYS724 compared to the other pedigree cohorts and the father–son based reference rate. Figure 1 presents the data for all cohorts for the 30 RMplex Y-STRs, while the data for all 49 Y-STRs, including the Yfiler Plus Y-STRs, are given in Table S1.

### Male relative differentiation rates

The male relative differentiation rate of a given set of Y-STRs refers to the rate at which a given pair of paternally related males (e.g., brothers, or first cousins) can be discriminated from each other by an allelic difference in at least one Y-STR marker. By taking advantage of the deep-rooted nature of a part of the pedigrees, we were able to establish differentiation rates for male relatives separated from one meiosis (i.e., separated by one generation: a father–son pair) up to 34 meioses. All RMplex data are presented in Table S2. Overall, by combining the results from all three cohorts, the set of RMplex Y-STRs achieved a differentiation rate of 43.3% for males separated by one meiosis, while males separated by two meioses (i.e., brothers and grandfather–grandson pairs) were differentiated in 66% of the cases. Moreover, relative differentiation for males separated by six or more meioses was over 95%, and male relatives that were twelve or more meioses apart were differentiated 100% of the time. Notably, the sample size of male relatives separated by one to thirteen meiosis was rather large with 334–966 pairs, while for those fourteen or more meiosis apart was markedly smaller (i.e., less than 100 pairwise comparisons).

**Fig. 2:**
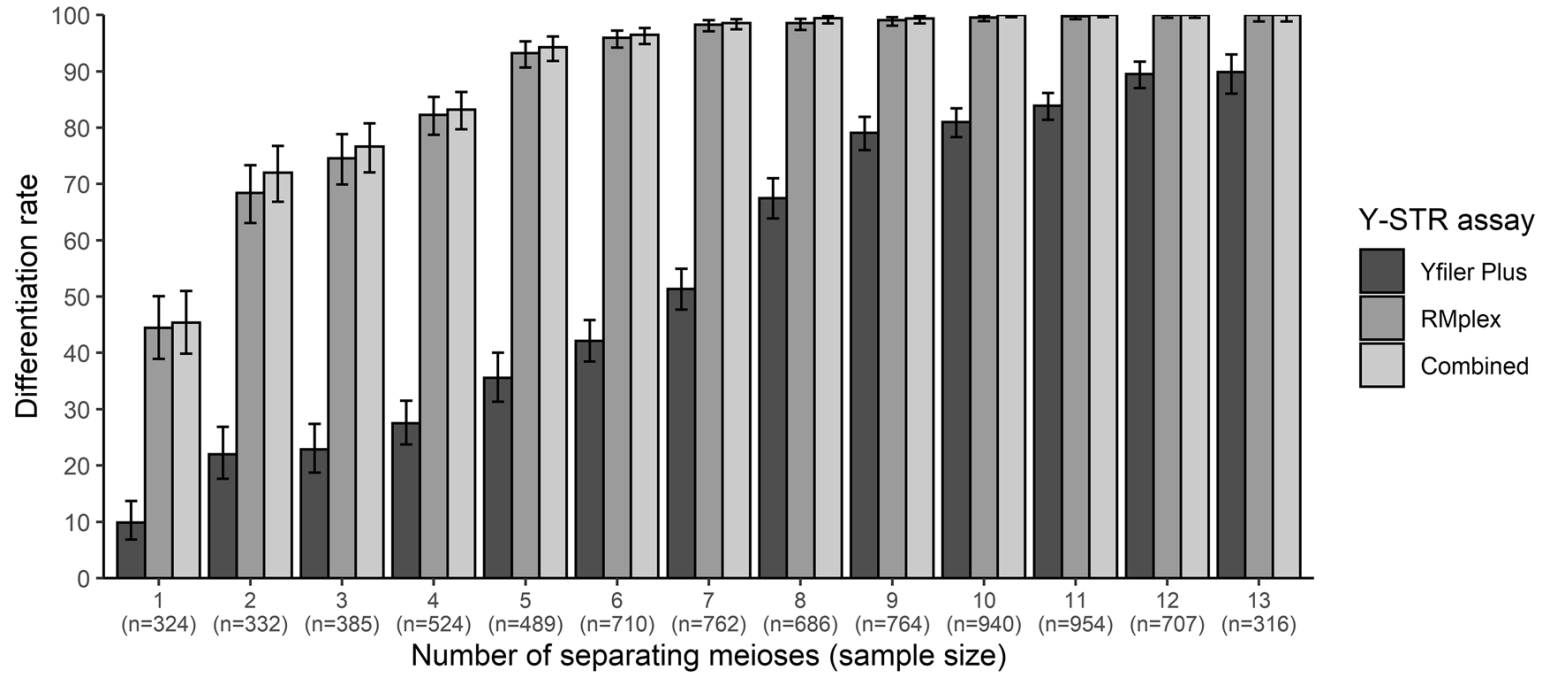
Male relative differentiation rates obtained from Cohort 1 pedigrees for RMplex (30 Y-STRs), Yfiler Plus (25 Y-STRs), and both assays combined (49 Y-STRs) for pairs of males related by 1–13 meioses. The error bars represent the 95% Clopper–Pearson intervals. Male relative differentiation is defined as a pair having at least one (but not excluding multiple) allelic differences

For Cohort 1, we describe the results in more detail because this cohort contains pedigrees that include a large number of different degrees of relatives, especially for previously understudied distantly related males up to 13 generations apart. Moreover, Cohort 1, additionally to RMplex, also has Yfiler Plus data available which allows for direct comparison between the set of Y-STRs with increased mutation rate in RMplex and a set containing mostly moderately mutating Y-STRs in Yfiler Plus, which allows linking the obtained findings with the underlying mutation rate of the markers used (Fig. 2, Table S3). This comparison highlighted that the set of RMplex Y-STRs was far superior to the set of Yfiler Plus Y-STRs in regards of the differentiation of both closely and more distantly related males (Fig. 2). With the set of Yfiler Plus Y-STRs, only 10% of the father–son pairs were differentiated, compared to 44% with the RMplex Y-STR set. Combining the markers from both assays only led to a marginal increase to 45% compared to RMplex Y-STRs alone. The differentiation rates increased with the number of meioses between two related males (Fig. 2), as was expected given the independent probabilities with which mutations occur during every meiosis that separates two relatives. The set of RMplex Y-STRs was able to differentiate over 95% of the male relatives separated by six meioses, while only 42% of such relatives were separated with Yfiler Plus Y-STRs. Complete differentiation of all relative pairs was achieved in men separated by twelve and more meioses using RMplex Y-STRs, by ten and more meioses using the combined assays, and never up to the thirteen meioses with Yfiler Plus Y-STRs. The Yfiler Plus Y-STR set had a maximum differentiation rate at 90% in males separated by 13 meioses, which was below the differentiation rates already achieved with the RMplex Y-STR set in males separated by five meioses.

**Fig. 3:**
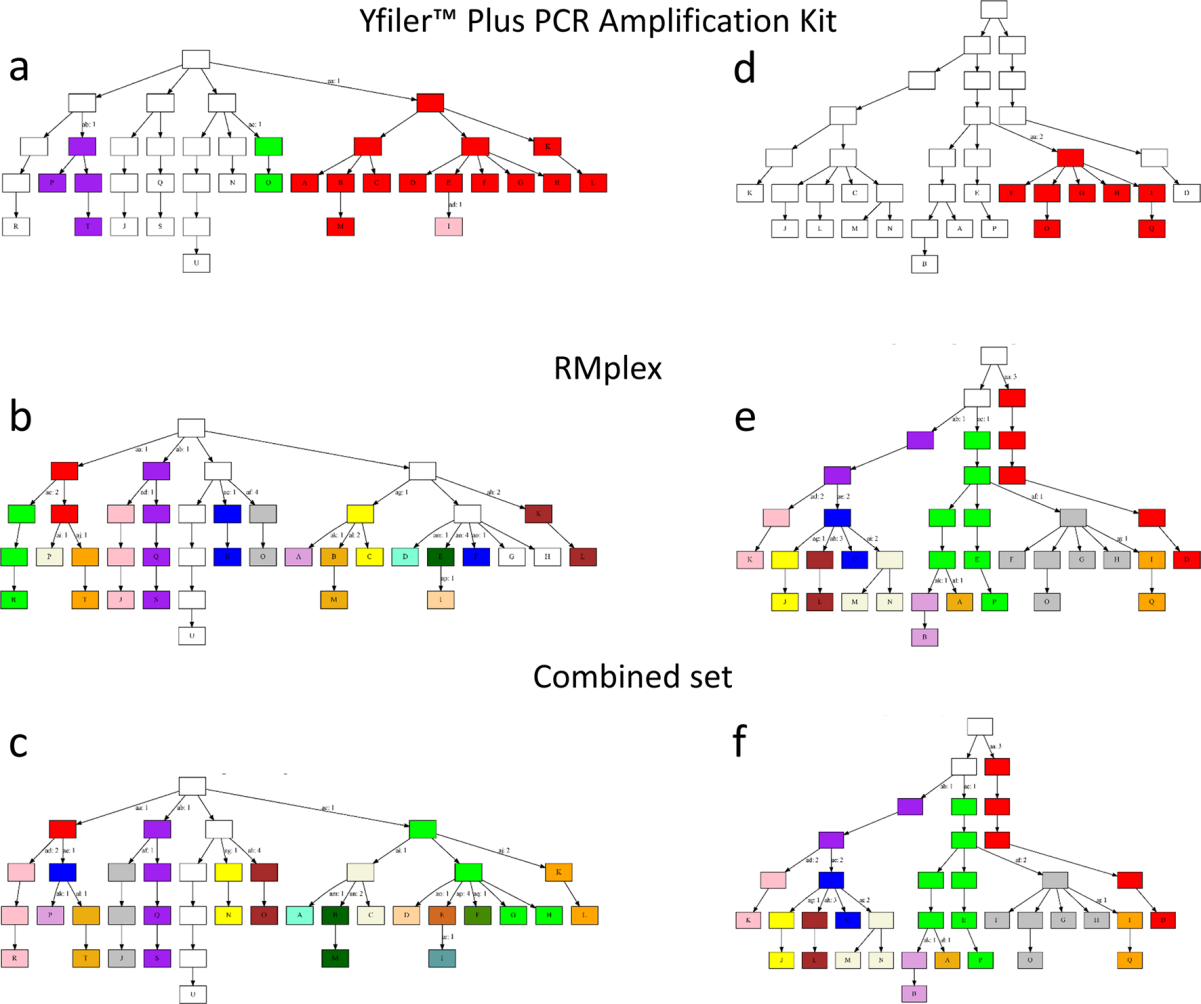
Male relative differentiation in two example pedigrees using Yfiler Plus (**a**, **d**), RMplex (**b**, **e**), and both assays combined (**c**, **f**). The different colors indicate unique haplotypes different from the inferred ancestral haplotype shown in white. The nodes with labels indicate individuals that were genotyped; individuals with unlabeled nodes were unavailable for genotyping. The colors in the unlabeled nodes indicate hypothetical haplotypes as the mutations could have occurred in any patrilineal ancestor that shares the color of the genotyped individual(s). The letters on the labels next to the arrows correspond to specific (sets of) mutations observed, whereas the numbers reflect the total number of mutational steps

To exemplify how the differences in differentiation rate between the two marker sets that largely differ by the underlying mutation rates affect the ability to differentiate individuals within a given pedigree, Fig. 3 shows two examples of pedigrees from Cohort 1. Figure 3a–c each shows a total of 21 genotyped individuals; using Yfiler Plus Y-STRs (Fig. 3a), a total number of five unique haplotypes was observed, including a single haplotype that uniquely identified a single individual. In the same pedigree using RMplex Y-STRs (Fig. 3b), the total number of haplotypes increased to fifteen, of which six were uniquely attributed to single individuals. By combining Y-STRs of both assays (Fig. 3c), a total of 17 haplotypes were observed of which seven could be attributed to single individuals. Figure 3d–f shows a similar pattern in a different pedigree.

### Prediction of patrilineal consanguinity

Next, we investigated if the observed differences in Y-STR genotype data between two related males can function as a reliable predictor for the degree of patrilineal consanguinity between those two males. To this end, we employed a multilayer perceptron classifier to develop models, which were trained on simulated data, that can predict the degree of patrilineal consanguinity (i.e., the number of separating meioses) based on the observed Y-STR allelic differences, i.e., mutations, between two related males, for RMplex Y-STRs and Yfiler Plus Y-STRs separately, as well as for the combined marker set. Figure S1 shows the results of those models for the scenario where no allelic differences were observed, i.e., a matching haplotype between the relatives, which would indicate a close relationship, particularly when many RM Y-STRs are included as with RMplex. Indeed, the 95% confidence interval for the set of RMplex Y-STRs ranged from one to six meioses. For Yfiler Plus Y-STRs, however, the 95% confidence interval was much wider, with one to 25 meioses, demonstrating a larger uncertainty about the relationship in the case of a matching Y-STR haplotype. When combining markers from both assays, the 95% interval remained one to six meioses; however, the cumulative probability (i.e., the sum of the probabilities obtained for each distance included in the interval) slightly increased from 95.5% with RMplex Y-STRs to 96.3% with both assays combined. Y-STR mutations are highly stochastic, as indicated by the high variance shown in Fig. S2. On average, the number of observed allelic differences increases the more distant the paternal familiar relationship is. As expected, for RMplex Y-STRs this trend was seen a lot stronger than for Yfiler Plus Y-STRs; while at the same time the variance observed with RMplex Y-STRs was larger. Generally, there was a strong overlap in the distribution of number of observed mutations between different meiotic distances, especially those in close proximity to one another.

**Fig. 4:**
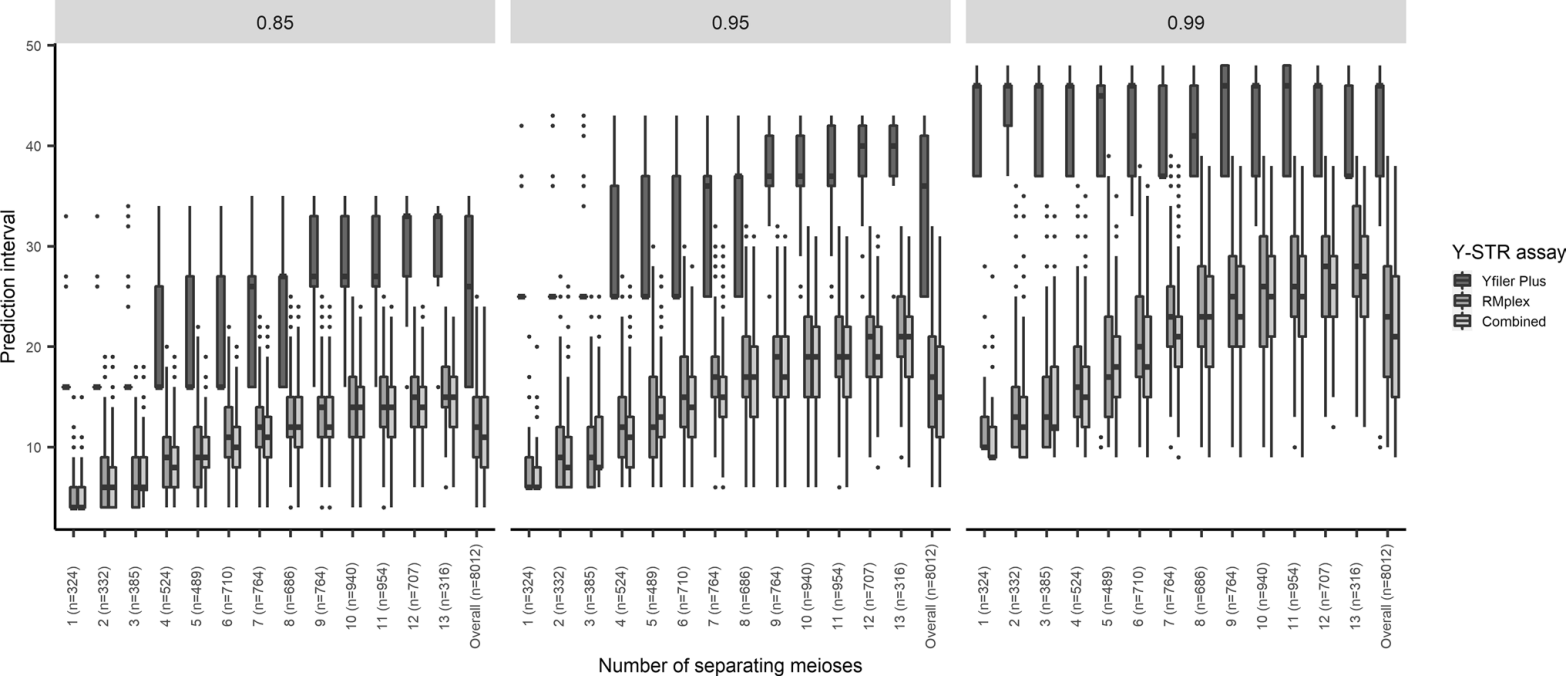
Boxplots showing the distribution of the prediction intervals of the three different multilayer perceptron classifiers trained to predict the degree of patrilineal consanguinity based on the observed mutations between pairs of paternally related males using Yfiler Plus, RMplex and both assays combined using three different predefined levels of confidence (85%, 95% and 99%)

**Fig. 5:**
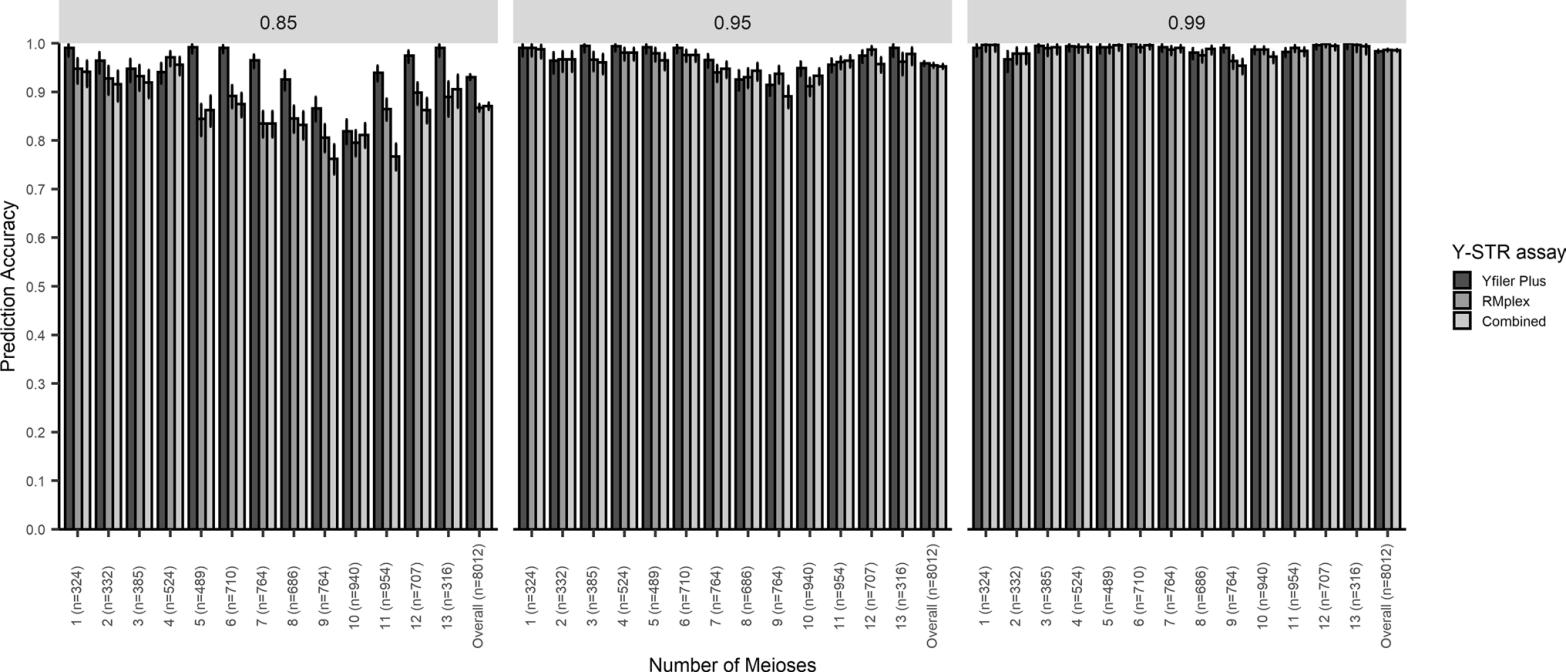
The accuracy of three different multilayer perceptron classifiers trained to predict the degree of patrilineal consanguinity based on the observed mutations between pairs of paternally related males using Yfiler Plus, RMplex, and both assays combined using three different predefined levels of confidence (85%, 95% and 99%). The error bars represent the 95% Clopper–Pearson intervals

To empirically demonstrate that indeed Y-STRs with a high mutation rate in RMplex are more suitable for the purpose of predicting patrilineal consanguinity compared to standard Y-STRs with lower mutation rates in Yfiler Plus, the newly developed models for Yfiler Plus Y-STRs, RMplex Y-STRs, and all Y-STRs from both assays combined were empirically tested on pairs of paternally related men of different degrees. To this end, we used the data from Cohort 1, because of the reasonably large sample size per each degree of relatedness being available in this cohort for male relatives separated by one to thirteen meioses ranging from 316 to 954 pairwise comparisons. Therefore, these thirteen generational groups were evaluated separately. Additionally, all pairs included in the cohort, including those separated by more than 13 meioses, were analyzed as a whole. The two most critical characteristics for predicting the degree of paternal relationship from the Y-STR data were evaluated: prediction accuracy (i.e., the percentage of pairs of which the true value fell within the prediction intervals) and precision (i.e., the size of the prediction intervals). The precision of when using mostly moderately mutating Y-STRs fell short of that obtained while using predominantly RM Y-STRs as indicated by the relatively large prediction intervals (Fig. 4). Another trend that became evident is that the size of the prediction intervals also increased in more distant relationships (Fig. 4). With regards to accuracy Yfiler Plus Y-STRs gave a slightly higher accurate predictions compared to RMplex Y-STRs and the combined Y-STRs. When looking at the overall prediction, i.e., including all levels of relationship, Yfiler Plus Y-STRs showed correct prediction in 93.0%, 95.8%, and 98.4%, for predefined confidence levels of 85%, 95%, and 99%, respectively. RMplex Y-STRs gave accurate prediction in 86.7%, 95.5%, and 98.6% for the same confidence levels, respectively; while the markers from both assays combined predicted accurately in 87.1%, 95.3%, and 98.5%, respectively (Fig. 5). The prediction accuracy was not constant among the different number of separating meioses, the accuracy of our models appears to be somewhat reduced in the proximity of nine meioses (Fig. 5). The models described here and a number of additional models for different (combinations of) Y-STRs kits that have not yet been empirically validated can be used through a web user interface on: ystr.erasmusmc.nl.

**Fig. 6:**
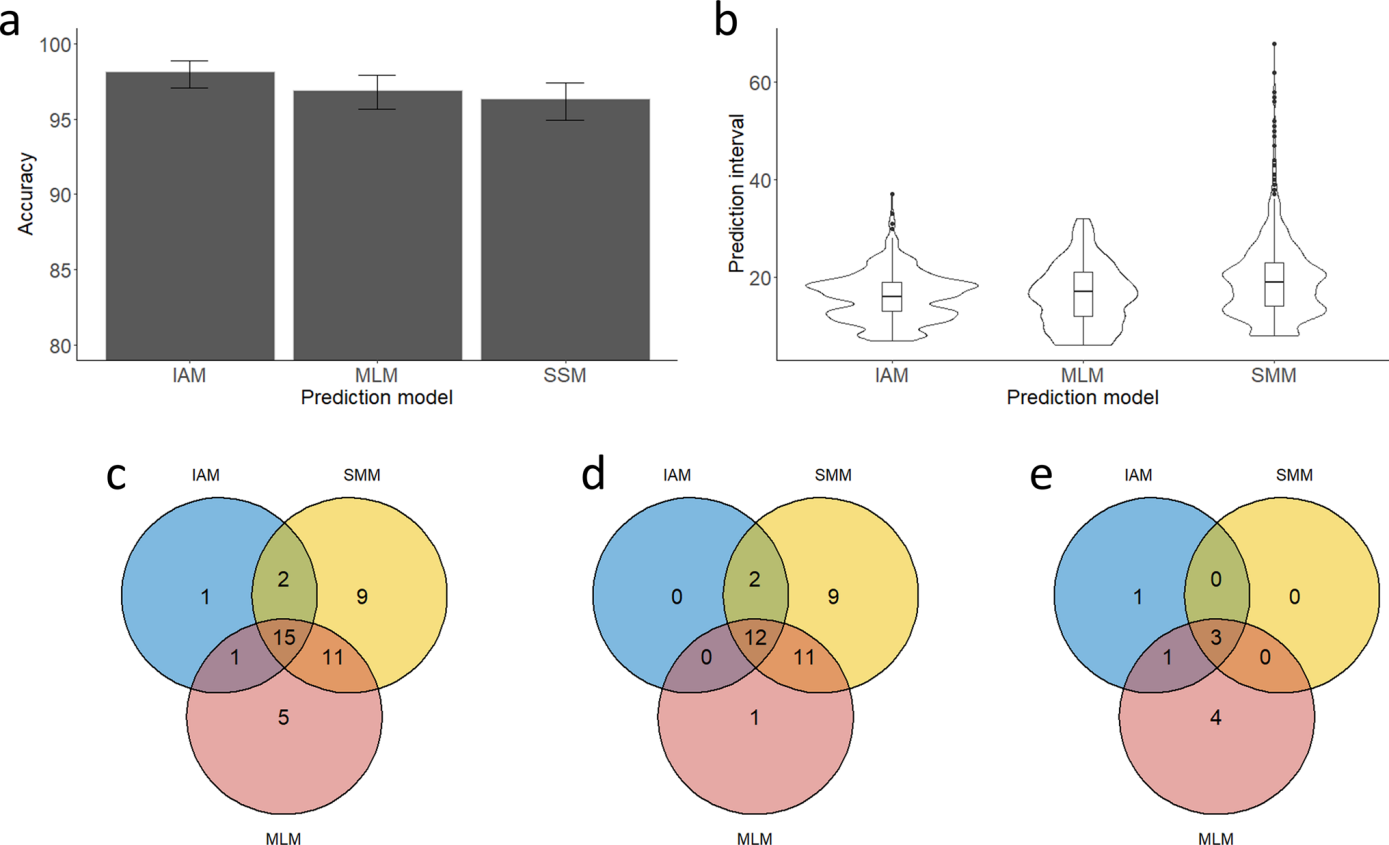
The performance of three different models to predict the degree of paternal consanguinity based on RMplex Y-STR data: Infinite Allele Model (IAM), Single Mutation Model (SMM) and the newly developed multilayer perceptron classifiers (MLM) using 95% confidence intervals. The performance was assessed by the accuracy (**a**), the precision (**b**). The recurrence of errors was further evaluated by using Venn diagrams showing the total number of errors (**c**), overestimations (**d**), and underestimations (**e**). Numbers in **c**–e reflect the total numbers out of the total of 1000 pairs that lead to incorrect predictions

To put the performance of our newly developed multilayer perceptron classifier (MLM) in perspective, we compared the results to two previously studied models to describe STR variations: the infinite alleles model (IAM) and the stepwise mutation model (SMM). All three models were evaluated by testing the same set of 1000 randomly selected pairs of paternally related men from all three cohorts. Notably, IAM outperformed the other two models both in regard of prediction accuracy (Fig. 6a) and precision (Fig. 6b); SMM, in turn, was the least well performing model out of the three. The accuracy of IAM was significantly higher than that of SMM (Fisher’s exact *p* value: 0.0204); the difference between IAM and MLM was not significant (*p* value: 0.1143), nor was the difference between SMM and MLM (*p* value: 0.5376). All three models delivered an accuracy > 95% (Fig. 6a), which was expected as the 95% confidence intervals were used by all models. To learn more about the nature of the prediction errors resulting from each of the three models, Venn diagrams were used for the total number of errors (Fig. 6c), the overestimations (Fig. 6d), and the underestimations (Fig. 6e). Overestimations were the most common type of prediction errors in each of the three models. Some pairs consistently lead to errors regardless of the model that was used. SMM and, to a slightly lesser degree, MLM overestimated the number of generations more often than IAM (Fig. 6d). Notably, SMM showed the lowest number of underestimations and in cases where it did, it was consistent with the other two models (Fig. 6e).

## Discussion

### Mutation rates

Previous RM Y-STR mutation rate studies mostly focused on father–son pairs. The advantage of such studies is that the degree of relationship, i.e., the number of separating meioses is known with certainty, e.g., because only father–son pairs with paternity confirmed with autosomal DNA from analyzing complete trio cases were used. Hence, from an observed Y-STR allelic differences between a father and his biological son, it can safely be concluded that a mutation had occurred. The disadvantage is that, unless very large numbers of father–son pairs are analyzed, the statistical power is low. Limited statistical power leads to limited reliability of the obtained mutation rate estimates. In addition to the costs and labor associated with typing such large number of samples, sample availability is also a limiting factor that needs to be overcome to perform accurate father–son pair based mutation rate studies.

Estimating mutation rates from pedigrees, on the other hand, comes with the advantage that, depending on the deep-rooting structure of the pedigree, many meiosis can be covered by analyzing only a restricted number of males. Thus, pedigree studies typically reach larger numbers of meioses, which theoretically allows for more reliable mutation rate estimates. The cost-effectiveness of using especially deep-rooting pedigrees can be striking. For example, in Cohort 2 of this study, a total of 2089 meioses were covered by analyzing only 265 individuals. To cover the same number of meioses using the father–son based approach would require genotyping almost 4200 individuals; ergo, 15-fold increased genotyping efforts and resources. However, the reliability of pedigree-based studies can be hampered by uncertainties caused by, for example, parallel mutations, back-forward mutations, and or multistep mutations (Claerhout et al. 2019, 2018). Furthermore, there can be uncertainty regarding the true biological relationship of the pairs (Larmuseau et al. 2013). Despite those uncertainties, in our study, 43 of the 49 Y-STRs we analyzed in total had pedigree-based mutation rate estimates that were not significantly different from the previous father–son based reference mutation rates that were established from fairly large numbers of father–son pairs.

Only six out of the 49 Y-STRs analyzed showed significantly different mutation rate estimates from the current pedigree data, compared to the previously obtained father–son based reference mutation rates. It is difficult to know the exact reason for these differences, which can be intrinsic to the methodology employed, or not. It is remarkable, that the three Y-STRs with higher mutation rate estimates in the current study all showed markedly higher absolute mutation rates compared to the three Y-STRs that displayed lower mutation rates in this study. The different mutation rate estimates could also be caused by stochastic effects, or could be a result of the different biogeographic origin of the subject of the different studies. As longer alleles (i.e., longest uninterrupted stretch, LUS) tend to mutate more frequently than their shorter counterparts and because different populations (i.e., different haplogroups) can exhibit different allelic distributions, some populations may exhibit remarkably high of low mutability for specific Y-STRs (Claerhout et al. 2018; Otagiri et al. 2022). The overall high concordance between father–son based and pedigree based mutation rates suggest that using pedigrees is a valid approach to estimate mutation rates.

### Male relative differentiation rates

We performed the most comprehensive study into male differentiation rates based on Y-STRs available to date, regarding the number of Y-STRs, the number of male relatives, and the number of degrees of paternal relationships we considered. These novel insights are expected to become highly relevant for future interpretation of Y-STR haplotypes derived from patrilineal relatives in all types of applications in anthropological studies, genealogical investigations and forensic casework. Previous studies mostly focused on less Y-STRs and only used close relatives such as father–sons and brothers, or only on a limited number of relatives separated by more generations (Adnan et al. 2016; Ambrosio et al. 2020; Ballantyne et al. 2012, 2014; Boattini et al. 2016; Javed et al. 2018; Neuhuber et al. 2022; Yuan et al. 2019; Zgonjanin et al. 2017).

The father–son differentiation rates of 10%, 44%, and 45% from Yfiler Plus Y-STRs, RMplex Y-STRs, and all Y-STRs, respectively (Fig. 2), that we obtained in the current pedigree study is comparable to the father–son differentiation rates of 14%, 42%, and 48%, respectively, previously established from father–son pairs for the same marker sets (Neuhuber et al. 2022). Another previous study (Ralf et al. 2020) hypothesized, based on differentiation rates estimated from mutation rates, that male relative differentiation rates of 99% would be achievable from eight meioses onwards, by using all 26 known RM Y-STRs. In the current pedigree study, we empirically showed that the 99% differentiation rate was reached with RMplex (including those 26 RM Y-STRs) from nine meioses onwards, closely agreeing with the previous theoretical expectation. Notably, our study is the first that demonstrates male relative differentiation in appreciable numbers for distant relatives separated by more than two meioses for the full set of 30 RMplex Y-STRs, previously only father–son pairs and a limited number of brothers were described (Neuhuber et al. 2022; Otagiri et al. 2022). Male relative differentiation of males separated by three to four meioses were only available for a subset of 13 RM Y-STRs (Adnan et al. 2016), and reliable data (i.e., with sufficient sample size) about Y-STR differentiation of males separated by more than four meioses was lacking completely. Overall, RMplex Y-STRs with increased mutation rate did fulfil their promise of delivering male relative differentiation with an unprecedented efficiency for all degrees of paternal relationships, as demonstrated.

The differentiation rates can provide forensic investigators with an expectation about the evidential value of a Y-STR haplotype match. Historically, the strongest value of Y-STRs in court cases has been to exclude a male suspect as being the donor of a crime scene stain. While, conversely a fully matching Y-STR haplotype was considered a non-exclusion. The state-of-the-art method to determine the value of a non-exclusion is through the use of population frequency databases such as YHRD (Roewer et al. 2020); the more frequently a Y-STR haplotype is observed in such databases, the lower the evidential value is regarded (Roewer et al. 2020). New generations of commercial Y-STR genotyping assays, such as Yfiler Plus contain more Y-STRs, including a limited number of RM Y-STRs, and have a much larger discrimination capacity resulting in the need for much larger databases. However, even in large frequency databases it can be expected that there will be many singletons (i.e., haplotypes observed only once in a population), or haplotypes that are not present in the database at all, because of its limited size relative to the whole population and given the diversity of the haplotypes (Caliebe et al. 2015). The differentiation rates obtained in this study show that, generally, only paternally related males separated by just a relatively low number of meioses share Y-STR haplotypes when using many RM Y-STRs. The high differentiation rates observed here, clearly show that the capacity to exclude potential crime scene sample donors that are related drastically improved when using RM Y-STRs rather than moderately mutating Y-STRs.

Moreover, the high differentiation rates of RM Y-STRs and RMplex provide a solution to genetic genealogist. With the tools that typically are at their disposal, i.e., Y-STRs, Y-SNPs, and autosomal DNA markers, it can be challenging to determine the correct position of an individual within a pedigree. RM Y-STRs, however, as can be asserted from the examples in Fig. 3, would allow to localize an individual’s position in a given pedigree with more precision. Furthermore, in anthropological genetics, in particular in population influenced by strong founder effects, male differentiation using RM Y-STRs can uncover population substructure when standard Y-STRs cannot because of high levels of homogeneity in the population. Lastly, the increased ability of RM Y-STRs to differentiate relatives may also be suitable to study recent migration events.

### Prediction of the degree of patrilineal consanguinity

Our results show that despite the stochastic nature of Y-STR mutations, it is feasible to predict the degree of patrilineal consanguinity of two males within a reasonably narrow range solely based on the number of observed Y-STR variations. We also showed that a higher precision (i.e., more narrow confidence intervals) could be achieved by analyzing Y-STRs with higher mutation rates compared to Y-STRs with moderate mutation rates, demonstrating the superiority of RM Y-STRs over moderately mutating Y-STRs also for this purpose. This latter finding is in agreement with a previous study that also found RM Y-STRs to deliver more precise estimations of the time since the most recent common ancestor (TMRCA) for other than forensic purposes (Boattini et al. 2019). Furthermore, we have shown that it is feasible to develop prediction models based on simulated Y-STR mutation data. The accuracy of the predictions based on our empirical data was largely in agreement with the expected accuracy based on the simulated data. The implication of these results is that such models can easily be developed for other sets of Y-STRs, given that the mutation rates of all markers in such a kit are known. In addition, multiple models could be built for the same sets of Y-STRs, based on different mutation rate estimates, for example if it is shown that the locus-specific mutation rates strongly differ in the population of interest. This method of investigation may become more precise over time as the number of addressable and well-characterized Y-STRs increases, for example by using massively parallel sequencing-based methods for data generation (Claerhout et al. 2021b).

In forensic genetics, genetic genealogy and also in anthropological studies where Y-STRs are applied, it is possible to encounter fully, or nearly matching Y-STR haplotypes, while other knowledge about the relationship of the two matching males is unavailable. However, even when using commercial Y-STR kits, such as Yfiler Plus that mostly contain moderately mutating Y-STRs, matching haplotypes can be detected in men that are distantly related and descendants of a male that lived many generations ago, as we demonstrated here (see Fig. 3). This also became apparent in Fig. S1a, where it was shown that the 95% confidence interval for a fully matching Yfiler Plus profile ranges from 1 to 25 meioses. Hence, even if a full Yfiler Plus Y-STR haplotype match was found between two males, this may only indicate that they share a common ancestor that dates back more than ten generations, i.e., several hundreds of years. In comparison, for RMplex Y-STRs with much higher mutation rate, the 95% interval ranges from one to six meioses. In cases when two males show a matching Yfiler Plus profile as the result of a distant common ancestor, RMplex would likely show multiple allelic variations and reflect the more distant relationship in the resulting prediction.

In our study, we found that the infinite alleles model (IAM) outperformed both stepwise mutation model (SMM) and the novel multilayer perceptron classifier (MLM) that we have introduced in the present study, although the differences were not striking. These results contradict a recent study by Claerhout et al*.* (2021a) which found SMM to outperform IAM, while in that study both methods delivered an accuracy that was well below the accuracy we found in the present study. This previous study also proposed a new method that was found to deliver more accurate results than IAM and SSM (Claerhout et al. 2021a). Unfortunately, we were unable to apply this newly proposed method, possibly due to the large number of RM Y-STRs included in our study leading to technical errors, potentially related to memory issues. Therefore, this method was not included in the comparison made here. Another study (Boattini et al. 2019), however, found IAM to be more accurate than SSM, which is in accordance with our results. The described accuracy in this latter study was higher than that described in the study from Claerhout et al*.* (2021a) for both models, but still lower than the accuracies that were achieved for IAM and SSM in the current study. A potential explanation for the reduced accuracy that was observed in both previous studies may be that both studies included more distantly related males, i.e., deep routed pedigrees; whereas the randomly drawn pairs in the present study predominantly were separated by one to thirteen meioses, as over 95% of our pairs were separated by meiotic distances in that range. Our data suggest that all models are valid and provide accurate predictions according to their confidence intervals. However, in our study IAM demonstrated a slightly better accuracy. The reason for this observation may be the relatively modest number of meioses that separated most of the thousand pairs that were used in our comparison. With a lower number of separating meioses, in general, not many mutations will have accumulated. In the case of RM Y-STRs, which also includes many multi-copy loci, however, some relatively closely related pairs may display multiple mutational steps in a multi-copy locus. SMM and MLM consider those as individual mutations, while IAM only considers two states: mutated or not-mutated. In principle this could explain the larger degree of overestimations as observed with SMM and MLM (Fig. 6d). In addition, the assumption that multi-step variations between pairs were the result of the result of multiple single-step mutations rather than a single multi-step mutation may have had an impact on rate of overestimations observed in SMM and MLM. More comprehensive future studies may shed more light on the differences that are observed between various studies, Y-STR kits and models.

Another model that would be interesting to further examine in the context of RM Y-STRs is the logistic mutation model (Jochens et al. 2011). Currently we lack sufficient data to fully evaluate this model on RM Y-STRs; moreover, the complex nature of many of the markers would favor sequencing data over fragment lengths. Nevertheless, this method of Y-STR mutation modelling could potentially result in more accurate and more precise predictions compared to the models evaluated here, as the logistic mutation model considers allele length, which is the largest driving force behind STR mutability.

### Conclusions

The study presented here shows that using pedigrees is an efficient approach to obtain empirical estimates of mutation rates and male relative differentiation rates for Y-STRs, including Y-STRs with increased mutation rates as studied here. We demonstrated that with RMplex a large proportion of closely and nearly all of distantly related males of different degrees of relationship can be differentiated, while much lower differentiation rates are achieved with the state-of-the-art commercial Y-STR kit Yfiler Plus. We show that predicting the degree of patrilineal consanguinity based on Y-STR data is feasible and that Y-STRs with high mutation rates such as those in RMplex delivered more precise prediction results than Y-STRs with lower mutation rates such as those in Yfiler Plus. Lastly, we emphasize that implementing new strategies involving Y-STRs with lower mutability and others with high mutability in routine forensic practice will open up new avenues to solve crimes that would otherwise remain unsolved.

## Materials and methods

### DNA samples

Within this study, a total of 2110 male DNA samples were analyzed, of these samples 64 were excluded because they showed too much variation (i.e., more than 10 variations) with other pedigree members to be reasonably considered to be truly patrilineally related. Another 253 samples were excluded from further analysis because of incomplete genotypic data, or because of the lack of other pedigree members with complete genotypic data. The remaining 1793 males were included in the subsequent analyses, these males belonged to a total of 403 pedigrees from three cohorts. Cohort 1 consisted of a total of 1075 Dutch males belonging to 201 male pedigrees. The samples included in Cohort 1 were collected in the context of the Erasmus Rucphen Family study (Sayed-Tabatabaei et al. 2005); in total Cohort 1 spanned 1856 meioses. Cohort 2 consisted of a total of 265 males belonging to 105 male pedigrees. All males in this cohort had either the Dutch or the Belgian nationality (the Belgian males all came from the Flemish part of Belgium); in total Cohort 2 spanned 2089 meioses. The larger cohort to which these samples belonged are described in more detail elsewhere (Larmuseau et al. 2019). Cohort 3 consisted of 453 males belonging to 97 pedigrees. All males in this cohort had the Pakistani nationality and had been part of a previous study into RM Y-STRs (Adnan et al. 2016); in total Cohort 3 spanned 405 meioses.

The different cohorts have different characteristics, where Cohort 2 consist mostly of males that share distant common paternal ancestors, Cohort 3 is characterized by containing closely related males. Cohort 1 contains pedigrees with large numbers of males with both recent and more distant common paternal ancestors, albeit not as distant as could be found in Cohort 2. Figure S3 visualizes the differences between the different cohorts with regard to the total number of male relative pairs and the degree of consanguinity between those pairs. Table 1 provides summary statistics that show the difference between the three cohorts.

**Table 1:** Summary statistics of the three cohorts included in this study

	Cohort 1	Cohort 2	Cohort 3
Individuals	1075	265	453
Number of pedigrees	201	105	97
Mean number of individuals per pedigree	5.4	2.5	4.7
Median number of individuals per pedigree	2	2	4
Max number of individuals per pedigree	50	16	10
Total meiosis covered	1856	2089	405
Mean number of meioses between pairs	7.86	17.51	2.29
Median number of meioses between pairs	8	17	2
Biogeographic ancestry	Northwestern Europe	Northwestern Europe	South Asian

### Y-STR Genotyping

All males were genotyped using RMplex for 30 Y-STRs with increased mutation rates under the conditions as described previously (Ralf et al. 2021), using the alternative primer for DYS570 and reducing the total reaction volume to 10 µL. Additionally, the males from Cohort 1 were also typed using Yfiler™ Plus PCR Amplification Kit (Thermo Fisher Scientific) following the manufacturer’s protocols, except for a reduced total reaction volume of 10 µL. All amplifications were performed on a Veriti™ 96-Well Fast Thermal Cycler (Thermo Fisher Scientific). Capillary electrophoreses were performed on a 3500 Series Genetic Analyzer (Thermo Fisher Scientific) equipped with a 36 cm 8-capillary array and using POP-4 (Thermo Fisher Scientific). GeneScan™ 600 LIZ™ dye Size Standard v2.0 (Thermo Fisher Scientific) was used as internal size standard. The interpretation of the electropherograms was performed using GeneMapper^®^ ID-X Software Version 1.5 (Thermo Fisher Scientific).

### Estimating mutation rates from pedigrees

To estimate the mutation rates using the pedigree information, we used the frequentist approach where the mutation rate was defined as the total number of observed mutations divided by the total number of meioses (notably, we did not use the pairwise meioses, which would result in counting the same meiosis several times, but the actual number of meioses that had occurred). This analysis was performed for each pedigree, the numbers of mutations and meioses from each pedigree were summed per cohort, and lastly the per-marker mutation rates were estimated by combining the three cohorts together. Clopper–Pearson intervals were used to indicate the uncertainty of the mutation rate estimates.

When estimating the number of mutations based on pedigree data (instead of father–son pairs) there is a need to make certain assumptions, as pedigrees may include males separated by many generations while the analyzed males only come from the more recent generations. The first assumption that was made, was that if no haplotypic difference was observed between a pair of males connected by individuals of which no data was available, that no mutation had occurred among all these males. The second assumption was that if multistep mutations were observed between two patrilineally related males, that this should be explained as multiple single step mutations rather than a single multistep mutation. The exception to the latter was in cases where the multi-step variation were found in a father–son pair, since in such cases a single multistep mutation was the only valid explanation. Furthermore, our approach always assumed the lowest number of mutations to explain the genotype variability between the individuals within a pedigree. These assumptions are expected to hold true in the majority of cases, but may lead to errors in some cases. Figure S4 shows an example of how the number of mutations were estimated in this study. In this example, a total of five mutations were concluded. Individual A–F shared the same mutation, which was most likely inherited from their most recent common ancestor; hence, these variations could be explained by a single mutation. Alternatively, the genotypes could be explained by three parallel mutations; however, observing the same mutations three times in three brothers (A–C) independently is highly unlikely and therefore this scenario was rejected. The same mutation was also observed in individual *N*; as this mutation is not shared by any of the close relatives of this individual, the most probable explanation is an independent mutation that took place in this individual. The other variation that was observed in this example pedigree was a mutation from allele 10 to allele 8 which was observed in two individuals. In individual *T* it could only be explained by a single two-step mutation, as there was also data available from the father of individual *T* (i.e., individual *Q*), where the mutation was not present. In individual *T*, however, there was no data available from the father or any other close paternal relative. Hence, for this individual it was assumed that two single-step mutations would be the most probable explanation; these mutations could have taken place at individual *U*, or at any of his three direct paternal ancestors. Importantly, the possibility that, just as in individual *T*, a single two-step mutation had taken place in one of these individuals cannot be ruled out based on the available data.

The most simple scenarios are encountered when dealing with single-copy Y-STRs, for example if one individual has allele 10 for a given Y-STR while a second individual from the same pedigree carries allele 12 for that same Y-STR, it will be assumed that two mutations had occurred. In contrast, multi-copy loci can lead to more complex scenarios; for example, in Fig. S5a the most straightforward solution (and the one that was assumed) is if allele 10 from individual A had mutated to allele 9 in individual B, so only one mutation had occurred. Alternatively, allele 10 from individual A could have mutated to allele 11 in individual B, while allele 11 in individual A mutated to allele 9 in individual B, this would require three mutational steps; although less likely such a scenario would not be impossible. Figure S5b shows a scenario where individual B carries a microvariant allele, while individual A does not. Here we considered the step from a microvariant allele to an adjacent conventional allele as one mutational step; hence, in Fig. S5b the mutation from allele 10 to 9.2 is considered as one mutation. In general, the scenario with the lowest number of mutation steps is preferred, Fig. S5c, however, shows an exception. If two individuals carried a microvariant allele, is was assumed that those two alleles are derived from the same copy; therefore in a situation as encountered in Fig. S5c, we would consider allele 11.2 to have mutated to 9.2 and allele 10 to 11, although mutations from allele 10 to 9.2 and from allele 11.2 to 11, respectively, would have explained the genotypes with less mutational steps. Lastly, Fig. S5d shows an example where two individuals have a different number of detected alleles in a multi-copy Y-STRs. For the genotyping we did not take peak heights into account for reasons explained elsewhere (Ralf et al. 2021), meaning that even if in individual B, allele 11 would show twice the height of allele 14, we would still call the genotype as 11, 14, instead of 11, 11, 14. In such cases too, the path with the lowest mutation steps is assumed, in this example that means that allele 10 in individual A would have likely mutated to allele 11 in individual B, ergo individual B would carry two copies of allele 11. The same logic as described above was applied in case a typically single-copy Y-STR would show a duplication in one of the individuals in a pedigree.

### Estimating differentiation rates

The frequentist approach was also used to calculate the male relative differentiation rates for every group of relatives separated by one to 34 meioses. Here, pairwise comparisons of all individuals within each pedigree were made, to identify all pairs of relatives that were separated by a certain number of meioses. From each pair separated by a given number of meioses the number of observed mutations between the individuals within the pair was assessed. The differentiation rate for given number of separating meioses (i.e., 1–34 in the total dataset) was calculated by dividing the number of pairs that displayed at least one allelic difference at one Y-STR marker, by the total number of pairs with that number of separating meioses. A comparative analysis between Yfiler Plus and RMplex was done on individuals from Cohort 1, as the sample size and the structure of the pedigrees in this cohort allowed to make a comprehensive assessment of the differentiation rate in a range of one to thirteen meioses. Clopper–Pearson intervals were estimated to indicate the statistical uncertainties of the differentiation rate estimates.

### Prediction of the degree of patrilineal consanguinity using a multilayer perceptron classifier

A machine learning based model (MLM), more specifically a multilayer perceptron classifier, was used to attempt to predict the number of meioses that separated a pair of relatives based on the observed Y-STR genotype differences. In order to train the models, data were simulated based on the reference mutation rate estimates for all Y-STRs derived from a recent study that combined data from many father–son based studies (Neuhuber et al. 2022). For each number of separating meioses in the range of 1–50, a total of 100,000 pairs were simulated (5 million data points in total per model). The probability of a mutation occurring at each individual Y-STR was set to be equal to the mutation rate. Once a mutation was simulated for a given Y-STR, the probability that it would mutate further in the next generation was half of the mutation rate, as was the probability that it would mutate back to the base position (i.e., no observed allelic difference between the pair for the given Y-STR). Moreover, the probability of a single two-step mutation occurring was set 3% of the total mutation probability. For multi-copy Y-STRs, each copy was simulated independently where the probability of a mutation occurring was equal to the mutation rate divided by the number of copies.

The simulated dataset was used to train models; the model used was a multilayer perceptron classifier as implemented by the python package scikit-learn (Pedregosa et al. 2011). We classified between one and 50 separating meioses based on a number of pre-determined sets of Y-STRs (Yfiler Plus, RMplex, and both assays combined). The model was trained using the default of 1 input layer, one hidden layer, one output layer, and otherwise, the default parameters for scikit-learns multilayer perceptron were used. The function randomizedSearchCV was used to randomly select the learning_rate, activation, alpha beta_1, beta_2, and the number of nodes in the hidden layer from a pre-defined feature space. In total 1000 different combinations of parameters where tested and each validated with a twofold cross validation step using the StratifiedKFold function of scikit-learn (Pedregosa et al. 2011).

The resulting models were validated using the empirical data generated in the context of this study. For each pair, and for each of the three Y-STR assays, the model assigned probabilities to each category, ranging from one to fifty separating meioses. Using those probabilities, prediction intervals were calculated at 85%, 95%, and 99% probability. These prediction intervals were determined by the cumulative probabilities obtained for each of the individual meiotic distances. To find the optimal prediction interval multiple iterations were performed, the size of the window was increased each iteration and then slid through all the possible combinations of adjacent meiotic distances (e.g., iteration#4: 1–4—> 2–5—> 3–6 (…)—> 47–50 meioses; iteration#5: 1–5—> 2–6—> 3–7 (…)—> 46–50 meioses). Once the predefined confidence level was exceeded using this approach the narrowest prediction interval (i.e., smallest window size) that resulted in the largest cumulative probability was returned as the prediction interval. The prediction accuracy of the models was determined by calculating the proportions of relative pairs where the true number of separating meioses fell within the respective predicted intervals. Additionally, to evaluate the precision of the different models, the size of the intervals was evaluated amongst the different assays and different number of separating meioses.

### Comparison with different prediction models

To compare the newly developed multilayer perceptron classifier based models with established models as described by Walsh (Walsh 2001), the R-script developed by Boattini et al*.* was implemented (Boattini et al. 2019). A random sub selection of a thousand pairs from the three cohorts was made (the distribution of different relationships is shown in Fig. S3). The number of mutational steps for those pairs were derived and used as input for SSM and MLM. The data had to be slightly modified where all non-zero values were transformed to the value 1 to serve as input for IAM. The R-script for IAM could be applied unmodified; however, SMM required a small modification as the high mutation rates found in RMplex led to errors. The numbers became bigger than the maximum floating point number in R of approximately 1.8e308. To overcome this error the “Rmpfr” packages (https://CRAN.R-project.org/package=Rmpfr) was used to allow for calculations up to 128 bit floating point numbers. The average mutation rate was derived from the same reference as used previously (Neuhuber et al. 2022) to match the mutation rates as used by MLM. The resulting 95% confidence intervals described the number of meioses to the common ancestor. Since MLM rather predicts the number of meioses separating the pair the intervals obtained from IAM and SSM were multiplied by a factor two. The lower point was rounded down and the upper bound was rounded up as the true number of separating meioses is always an integer.

### Data visualization

Plots of pedigree structures were made using yEd (https://www.yworks.com/products/yed). Graphs were made using Rstudio in combination with the “ggplot2” packages (Wickham 2011). Venn diagram were made in Rstudio using the “ggven” packages. The probability graphs in Fig. S1 were made using the online tool presented in this publication which can be found on ystr.erasmusmc.nl.

## Supplementary Information

Below is the link to the electronic supplementary material.

Supplementary file1 (PDF 1530 kb)

## Data Availability

The datasets generated during and/or analyzed during the current study are not made publicly available to maintain the anonymity of the participants, but are available from the corresponding author on reasonable request.
