# Supplementary material for: Large-scale pedigree analysis highlights rapidly mutating Y-chromosomal short tandem repeats for differentiating patrilineal relatives and predicting their degrees of consanguinity
Source: Hum Genet. 2022 Oct 3;142(1):145–60. doi: 10.1007/s00439-022-02493-2 (PMC9839801; doi:10.1007/s00439-022-02493-2)
Supplement: Supplementary file 1 — Supplementary file1 (PDF 1530 kb) [file 439_2022_2493_MOESM1_ESM.pdf]

***Human Genetics*, Supporting information to:**

Large-scale pedigree analysis highlights rapidly mutating Y-chromosomal short tandem repeats for differentiating patrilineal relatives and predicting their degrees of consanguinity

Arwin Ralf[1*], Diego Montiel González[1] Dion Zandstra[1], Bram van Wersch[1], Nefeli Kousouri[1], Peter de Knijff[2], Atif Adnan[3], Sofie Claerhout[4,5], Mohsen Ghanbari[6], Maarten H.D. Larmuseau[7,8,9], Manfred Kayser[1*]

[1] Department of Genetic Identification, Erasmus MC, University Medical Center Rotterdam, Rotterdam, the Netherlands

[2] Forensic Laboratory for DNA Research, Department of Human Genetics, Leiden University Medical Centre, Leiden, The Netherlands

[3] Department of Forensic Sciences, College of Criminal Justice, Naif Arab University of Security Sciences, Riyadh, Saudi Arabia

[4] Forensic Biomedical Sciences, Department of Imaging & Pathology, KU Leuven, Leuven, Belgium

[5] Interdisciplinary Research Facility Life Sciences, KULAK Campus Kortrijk, Kortrijk, Belgium

[6] Department of Epidemiology, Erasmus MC, University Medical Center Rotterdam, Rotterdam, the Netherlands

[7] Laboratory of Human Genetic Genealogy, Department of Human Genetics, KU Leuven, Leuven, Belgium

[8] ARCHES - Antwerp Cultural Heritage Sciences, Faculty of Design Sciences, University of Antwerp, Antwerp, Belgium

[9] Histories vzw, Gent, Belgium

* Corresponding author email: m.kayser@erasmusmc.nl (MK) or a.ralf@erasmusmc.nl (AR)

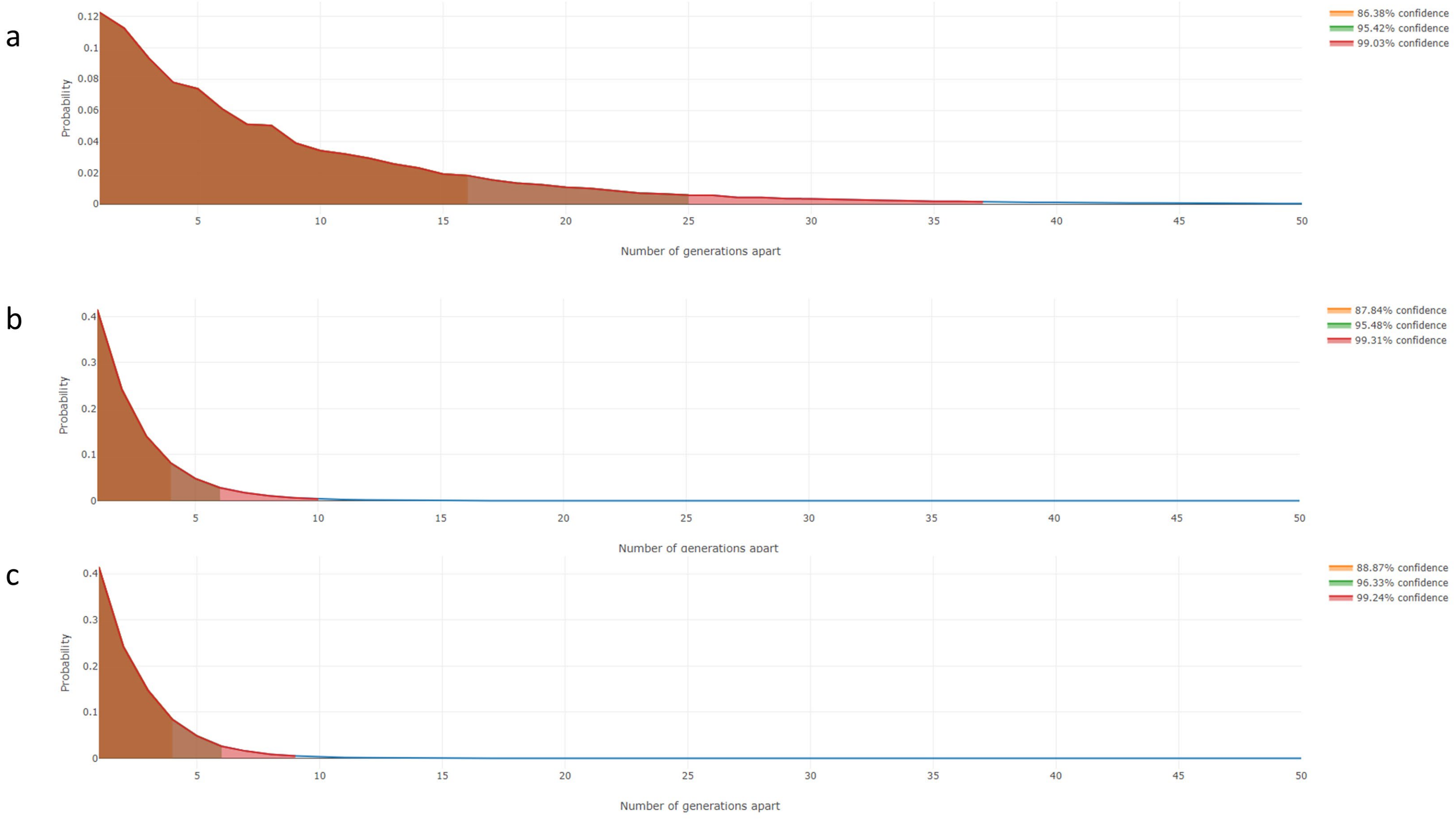

Fig S1: Predicted intervals with at least 85%, 95% and 99% probability with Yfiler Plus (a), RMplex (b) and the two assays combined (c) when no mutations between two individuals are observed.

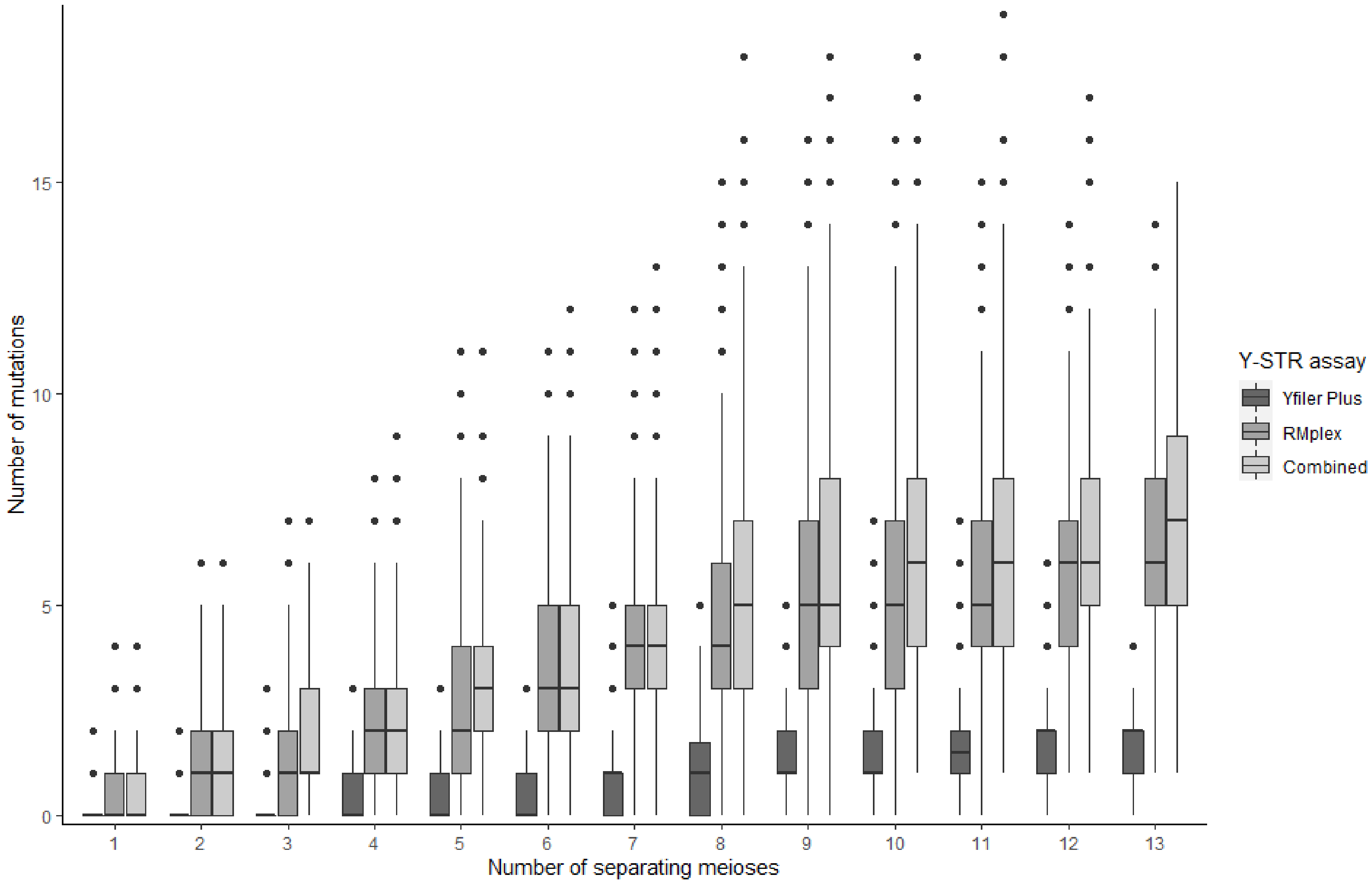

Fig S2: Boxplots showing the accumulation of mutations with increasing numbers of meioses.

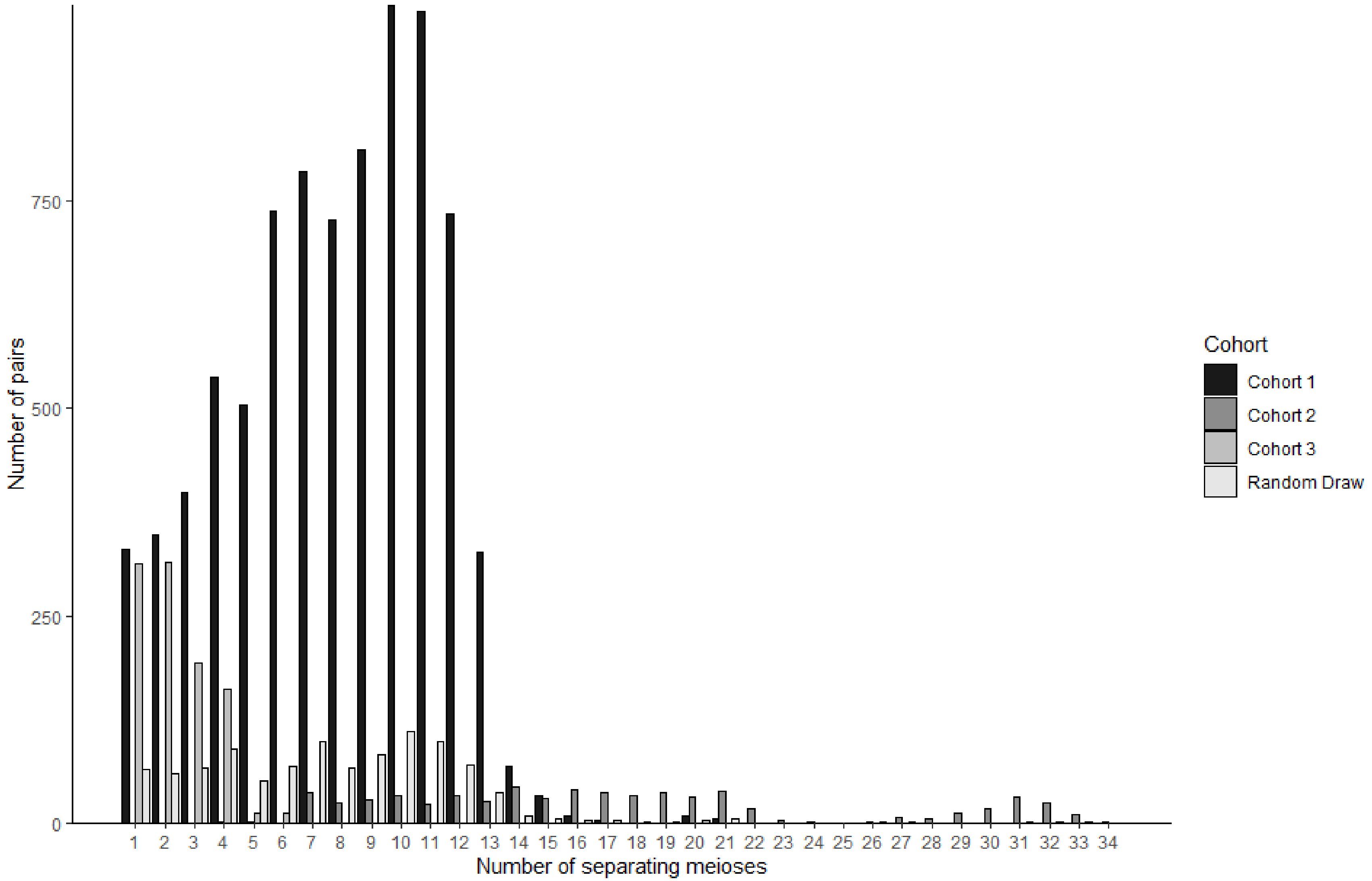

Fig S3: Distribution of number of separating meioses for all pairs of males in the different cohorts and for the 1000 randomly drawn pairs for the prediction model comparisons.

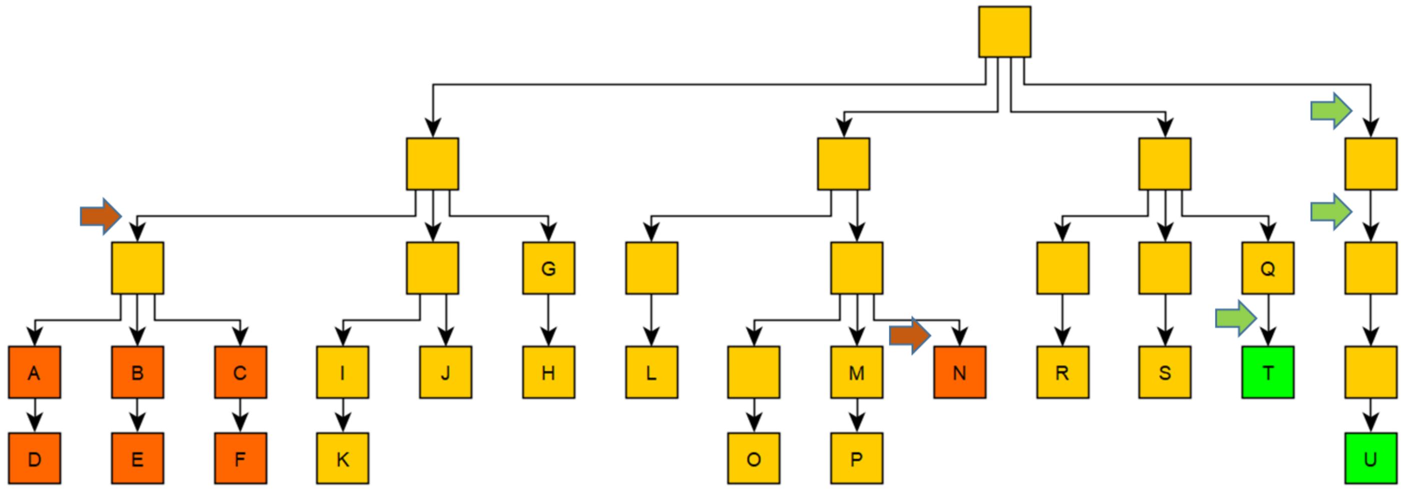

Individual	Genotype
A	11
B	11
C	11
D	11
E	11
F	11
G	10
H	10
I	10
J	10
K	10
L	10
M	10
N	11
O	10
P	10
Q	10
R	10
S	10
T	8
U	8

Fig S4: Example of a pedigree spanning 35 meioses, where a total of five mutations were estimated based on the observed genotype variations.

a.

Indv. A	Indv. B: 9	11	16
10	-1	+1	+6
11	-2	0	+5
16	-7	-5	0

b.

Indv. A	Indv. B: 9.2	11	16
10	-1	+1	+6
12	-3	-1	+4
16	-7	-5	0

c.

Indv. A	Indv. B: 9.2	11	16
10	-1	+1	+6
11.2	-2	-1	+5
16	-7	-5	0

d.

Indv. A	Indv. B: 11	14
10	+1	+4
11	0	+3
16	-5	-2

Fig S5: Various scenarios that could be encountered when dealing with multi-copy loci, the solutions that our approach preferred in these cases are highlighted.

Table S1: Combined mutation rate estimates as derived from all pedigreed included within the three analyzed cohorts.

Marker	Total meioses	Mutations	Mutation rate ($x10^{-3}$)	95% confidence range ($x10^{-3}$)	Reference value [1] ($x10^{-3}$)	Fisher's exact p-value
DYF399S1	4354	305	70.1	62.6 - 78.0	62.8	0.125
DYF1001	4354	233	53.5	47.0 - 60.6	48.0	0.372
DYF1000	4354	211	48.5	42.3 - 55.3	35.9	**0.018**
DYF403S1a	4354	168	38.6	33.1 - 44.7	27.3	**0.001**
DYS724	4354	167	38.4	32.8 - 44.5	48.0	0.074
DYS711	4354	131	30.1	25.2 - 35.6	26.6	0.479
DYS712	4354	113	26.0	21.4 - 31.1	31.1	0.221
DYS612	4354	99	22.7	18.5 - 27.6	16.3	**0.012**
DYS1012	4354	97	22.3	18.1 - 27.1	15.8	0.091
DYR88	4354	94	21.6	17.5 - 26.4	26.3	0.253
DYS1010	4354	84	19.3	15.4 - 23.8	14.0	0.133
DYF1002	4354	78	17.9	14.2 - 22.3	16.8	0.841
DYS518	4354	71	16.3	12.8 - 20.5	13.3	0.152
DYF404S1	4354	61	14.0	10.7 - 18.0	12.5	0.509
DYS547	4354	60	13.8	10.5 - 17.7	14.7	0.751
DYS627	4354	59	13.6	10.3 - 17.4	14.5	0.709
DYS1007	4354	59	13.6	10.3 - 17.4	17.2	0.274
DYS526b	4354	57	13.1	9.9 - 16.9	12.3	0.735
DYS713	4354	53	12.2	9.1 - 15.9	13.9	0.555
DYS626	4354	50	11.5	8.5 - 15.1	8.6	0.122
DYS449	4354	45	10.3	7.5 - 13.8	11.2	0.673
DYS570	4354	44	10.1	7.4 - 13.5	8.3	0.295
DYF393S1	4354	42	9.6	7.0 - 13	7.1	0.332
DYS1003	4354	41	9.4	6.8 - 12.8	12.6	0.245
DYS576	4354	40	9.2	6.6 - 12.5	12.7	0.072
DYF387S1	4354	37	8.5	6.0 - 11.7	10.2	0.363
DYS458	1860	15	8.1	4.5 - 13.3	8.5	1.000
DYF403S1b	4354	30	6.9	4.7 - 9.8	9.1	0.243
DYS1005	4354	26	6.0	3.9 - 8.7	9.8	0.118
DYS460	1860	11	5.9	3.0 - 10.6	4.3	0.343
DYS1013	4354	21	4.8	3.0 - 7.4	10.8	**0.009**
DYS385	1860	8	4.3	1.9 - 8.5	7.5	0.137
DYS390	1860	8	4.3	1.9 - 8.5	2.7	0.245
DYS391	1860	7	3.8	1.5 - 7.7	2.5	0.328
DYS389II	1860	7	3.8	1.5 - 7.7	5.5	0.487
DYS439	1860	7	3.8	1.5 - 7.7	4.8	0.713
DYS442	4354	15	3.4	1.9 - 5.7	7.4	**0.047**
DYS448	1860	6	3.2	1.2 - 7.0	0.8	**0.016**
DYS533	1860	6	3.2	1.2 - 7.0	3.5	1.000
DYS635	1860	5	2.7	0.9 - 6.3	3.8	0.541
YGATAH4	1860	4	2.2	0.6 - 5.5	1.9	0.777
DYS389I	1860	4	2.2	0.6 - 5.5	2.4	1.000
DYS456	1860	3	1.6	0.3 - 4.7	4.4	0.108
DYS481	1860	3	1.6	0.3 - 4.7	4.7	0.070
DYS19	1860	3	1.6	0.3 - 4.7	2.0	1.000
DYS438	1860	2	1.1	0.1 - 3.9	0.3	0.142
DYS393	1860	1	0.5	0.0 - 3.0	1.7	0.346
DYS437	1860	1	0.5	0.0 - 3.0	1.2	0.710
DYS392	1860	0	0.0	0.0 - 2.0	0.8	0.621

[1] the reference values are the consensus mutation rate estimates from: Franz Neuhuber, et al.,
Improving the differentiation of closely related males by RMplex analysis of 30 Y-STRs with high mutation rates,
Forensic Sci Int Genet. 2022 May;58:102682

Table S2: Observed differentiation rates using RMplex in three different cohorts and all three cohorts combined.

	Cohort 1			Cohort 2			Cohort 3			Overall		
**Meioses**	**Number of pairs**	**Differentiated (%)**	**Mean mutations (SD)**	**Number of pairs**	**Differentiated (%)**	**Mean mutations (SD)**	**Number of pairs**	**Differentiated (%)**	**Mean mutations (SD)**	**Number of pairs**	**Differentiated (%)**	**Mean mutations (SD)**
1	324	144 (44.4)	0.6 (0.8)	0			313	132 (42.2)	0.6 (1.0)	637	276 (43.3)	0.6 (0.9)
2	332	227 (68.4)	1.2 (1.2)	0			315	200 (63.5)	1.1 (1.3)	647	427 (66.0)	1.2 (1.2)
3	385	287 (74.5)	1.6 (1.5)	0			193	154 (79.8)	1.5 (1.2)	578	441 (76.3)	1.6 (1.4)
4	524	431 (82.3)	1.9 (1.5)	1	1 (100)	2 (0)	161	141 (87.6)	1.8 (1.2)	686	573 (83.5)	2.1 (2.3)
5	489	456 (93.3)	2.8 (1.8)	1	1 (100)	7 (0)	13	13 (100)	1.6 (1.1)	503	470 (93.4)	2.8 (2.1)
6	710	681 (95.9)	3.4 (2.0)	0			12	12 (100)	1.2 (0.4)	722	693 (96.0)	3.6 (2.9)
7	762	749 (98.3)	4.0 (2.2)	23	22 (95.7)	4.8 (2.1)				785	771 (98.2)	4.1 (2.6)
8	686	676 (98.5)	4.6 (2.5)	15	15 (100)	5.2 (3.0)				701	691 (98.6)	4.9 (3.3)
9	764	757 (99.1)	5.1 (2.8)	16	16 (100)	5.5 (3.1)				780	773 (99.1)	5.3 (3.5)
10	940	936 (99.6)	5.3 (2.6)	16	16 (100)	12.4 (17.2)				956	952 (99.6)	5.7 (4.1)
11	954	952 (99.8)	5.4 (2.4)	12	12 (100)	7.4 (3.4)				966	964 (99.8)	5.6 (3.0)
12	707	707 (100)	5.8 (2.3)	23	23 (100)	7.7 (2.2)				730	730 (100)	6.0 (2.8)
13	316	316 (100)	6.3 (2.3)	18	18 (100)	8.3 (2.6)				334	334 (100)	6.6 (2.8)
14	67	67 (100)	6.6 (2.3)	16	16 (100)	8.3 (2.9)				83	83 (100)	6.9 (2.5)
15	34	34 (100)	7.9 (1.4)	12	12 (100)	7.9 (3.4)				46	46 (100)	7.9 (2.1)
16	8	8 (100)	8.6 (1.3)	25	25 (100)	10.1 (2.7)				33	33 (100)	9.7 (2.5)
17	4	4 (100)	9.3 (0.8)	23	23 (100)	10.5 (5.0)				27	27 (100)	10.3 (4.6)
18				22	22 (100)	8.8 (3.1)				22	22 (100)	8.8 (3.1)
19				20	20 (100)	8.9 (3.2)				20	20 (100)	8.9 (3.2)
20	9	9 (100)	14 (1.2)	16	16 (100)	8.1 (3.6)				25	25 (100)	10.2 (4.1)
21	5	5 (100)	14.2 (1.5)	24	24 (100)	11.4 (4.4)				29	29 (100)	11.9 (4.2)
22				10	10 (100)	14.7 (3.7)				10	10 (100)	14.7 (3.7)
23				3	3 (100)	15.7 (4.0)				3	3 (100)	15.7 (4.0)
24				1	1 (100)	8 (0)				1	1 (100)	8 (0)
25												
26				1	1 (100)	14 (0)				1	1 (100)	14 (0)
27				6	6 (100)	15.2 (3.7)				6	6 (100)	15.2 (3.7)
28				3	3 (100)	14.7 (2.6)				3	3 (100)	14.7 (2.6)
29				2	2 (100)	19.5 (4.5)				2	2 (100)	19.5 (4.5)
30				6	6 (100)	13.7 (2.8)				6	6 (100)	13.7 (2.8)
31				14	14 (100)	13.8 (2.9)				14	14 (100)	13.8 (2.9)
32				15	15 (100)	13.1 (1.6)				15	15 (100)	13.1 (1.6)
33				7	7 (100)	13.9 (1.8)				7	7 (100)	13.9 (1.8)
34				1	1 (100)	16 (0)				1	1 (100)	16 (0)

Table S3: Direct comparison of Yfiler Plus, RMplex and both assays combined on differentiation of males separated by 1-13 meioses in Cohort 1

Assay	Meioses	Pairs	Differentiated	Differentiation rate (%)	Clopper-Pearson CI lower bound	Clopper-Pearson CI upper bound
Yfiler Plus	1	324	32	9.88	6.85	13.66
Yfiler Plus	2	332	73	21.99	17.65	26.83
Yfiler Plus	3	385	88	22.86	18.76	27.38
Yfiler Plus	4	524	144	27.48	23.7	31.52
Yfiler Plus	5	489	174	35.58	31.34	40.01
Yfiler Plus	6	710	299	42.11	38.45	45.84
Yfiler Plus	7	762	391	51.31	47.7	54.92
Yfiler Plus	8	686	463	67.49	63.85	70.99
Yfiler Plus	9	764	604	79.06	76	81.89
Yfiler Plus	10	940	761	80.96	78.3	83.42
Yfiler Plus	11	954	800	83.86	81.37	86.14
Yfiler Plus	12	707	633	89.53	87.04	91.69
Yfiler Plus	13	316	284	89.87	86.01	92.97
RMplex	1	324	144	44.44	38.95	50.04
RMplex	2	332	227	68.37	63.07	73.34
RMplex	3	385	287	74.55	69.89	78.82
RMplex	4	524	431	82.25	78.71	85.43
RMplex	5	489	456	93.25	90.65	95.31
RMplex	6	710	681	95.92	94.19	97.25
RMplex	7	762	749	98.29	97.1	99.09
RMplex	8	686	676	98.54	97.34	99.3
RMplex	9	764	757	99.08	98.12	99.63
RMplex	10	940	936	99.57	98.91	99.88
RMplex	11	954	952	99.79	99.24	99.97
RMplex	12	707	707	100	99.48	100
RMplex	13	316	316	100	98.84	100
Combined	1	324	147	45.37	39.86	50.97
Combined	2	332	239	71.99	66.82	76.75
Combined	3	385	295	76.62	72.07	80.76
Combined	4	524	436	83.21	79.72	86.31
Combined	5	489	461	94.27	91.83	96.16
Combined	6	710	685	96.48	94.85	97.71
Combined	7	762	751	98.56	97.43	99.28
Combined	8	686	682	99.42	98.51	99.84
Combined	9	764	759	99.35	98.48	99.79
Combined	10	940	940	100	99.61	100
Combined	11	954	954	100	99.61	100
Combined	12	707	707	100	99.48	100
Combined	13	316	316	100	98.84	100
